# Analysis of Monensin Sensitivity in *Toxoplasma gondii* Reveals Autophagy as a Mechanism for Drug Induced Death

**DOI:** 10.1371/journal.pone.0042107

**Published:** 2012-07-25

**Authors:** Mark D. Lavine, Gustavo Arrizabalaga

**Affiliations:** Department of Biological Sciences, University of Idaho, Moscow, Idaho, United States of America; University of Oklahoma Health Sciences Center, United States of America

## Abstract

Understanding the mechanisms by which anti-parasitic drugs alter the physiology and ultimately kill is an important area of investigation. Development of novel parasitic drugs, as well as the continued utilization of existing drugs in the face of resistant parasite populations, requires such knowledge. Here we show that the anti-coccidial drug monensin kills *Toxoplasma gondii* by inducing autophagy in the parasites, a novel mechanism of cell death in response to an antimicrobial drug. Monensin treatment results autophagy, as shown by translocation of ATG8 to autophagosomes, as well as causing marked morphological changes in the parasites' mitochondria. Use of the autophagy inhibitor 3-methyladenine blocks autophagy and mitochondrial alterations, and enhances parasite survival, in monensin-exposed parasites, although it does not block other monensin-induced effects on the parasites, such as late S-phase cell cycle arrest. Monensin does not induce autophagy in a parasite strain deficient in the mitochondrial DNA repair enzyme TgMSH-1 an enzyme that mediates monensin-induced late S-phase arrest. TgMSH-1 therefore either mediates cell cycle arrest and autophagy independently, or autophagy occurs downstream of cell cycle arrest in a manner analogous to apoptosis of cells arrested in G_2_ of the cell cycle. Overall, our results point to autophagy as a potentially important mode of cell death of protozoan parasites in response to antimicrobial drugs and indicate that disruption of the autophagy pathway could result in drug resistance.

## Introduction

Development of novel anti-parasitic drugs depends on having an in depth understanding of the mechanisms by which such drugs alter the physiology and ultimately kill the parasite [1]. This is particularly important for apicomplexan parasites, which include the causative agents for such important diseases as malaria (*Plasmodium*), toxoplasmosis (*Toxoplasma*), coccidiosis (*Eimeria*), and cryptosporidiosis (*Cryptosporidium*). All these parasites are of critical importance to human or animal health, and all have shown the development of resistance to currently available drugs [2]–[5]. In the analysis of drug mode of action, focus has traditionally been on the molecular and cellular processes affected by the drug. Nonetheless, little is known about how the parasites actually die as a consequence of the inhibitory effects of the drug. Such analysis could reveal pathways that when induced lead to parasite death and which also can lead to drug resistance when disrupted.

One of the most important classes of antiparasitic agents are ionophores, which have been used more widely in veterinary medicine than any other medicinal agents [6]. We have thus investigated the mode of action of one such widely used ionophore, monensin, a broad-spectrum antimicrobial that has been shown to be effective against a number of apicomplexan parasites, including members of the genera *Plasmodium*, *Toxoplasma*, and *Eimeria* [2], [7], [8]. We recently demonstrated that monensin causes cell cycle arrest of *Toxoplasma gondii* in late S-phase of the cell cycle, and that null mutations in a mitochondrial MutS homologue (TgMSH-1) abrogate this cell cycle arrest and provide the parasites with resistance to monensin [9], [10]. This S-phase arrest was not a general stress response, and did not cause activation of genes specific to the latent form, unlike other stressors such as exposure to elevated temperature (Lavine and Arrizabalaga, 2011).

Our results led us to formulate a model for the mode of action of monensin on *T. gondii*, and possible mechanism for TgMSH-1-mediated resistance. We hypothesize that a direct or indirect stress on the parasite's mitochondrion activates a TgMSH-1 dependent signaling cascade that results in late S-phase arrest, akin to the G_2_ arrest mediated by MSH molecules from other cell types [11], [12]. Cells that are arrested at checkpoints such as G_2_ in which damaged structures, particularly DNA, cannot be repaired, either due to the severity of the damage or continued presence of the damaging agent, typically die through the process of apoptosis [13]. Thus our model for the action of monensin similarly predicted that *T. gondii* exposed to monensin die as a result of prolonged arrest at a late S-phase checkpoint. However, we have found no evidence of apoptosis in *T. gondii* as a result of monensin exposure, and evidence for the presence of an apoptotic pathway in *T. gondii* is currently lacking. Instead we explored evidence for autophagy being the cell death pathway induced by monensin in *T. gondii*.

Recently, several reports have shown induction of autophagy in *T. gondii* in response to nutrient stress: incubation of extracellular parasites in saline solution (Hank's buffered salt solution – HBSS) [14] or intracellular parasites in cell culture medium diluted with HBSS [15]. In addition to its function in cell survival during stress conditions, autophagy can act as a distinct method of cell death, termed autophagic cell death [16]. Thus we investigated whether autophagy could play a role analogous to that of apoptosis in other types of cells after cell cycle arrest. Here we present evidence that monensin induces autophagy of *T. gondii*, that this process can result in the death of the parasite, and that monensin-induced autophagy is downstream of events dependent on the function of the TgMSH-1 enzyme.

## Materials and Methods

### Parasite and host strain maintenance and reagents

RHΔ*hpt* strain *T. gondii*, which lack a functional hypoxanthine-xanthine-guanine phosphoribosyltransferase (*hpt*) gene, were maintained by passage through human foreskin fibroblasts (HFFs) at 37°C and 5% CO_2_. HFFs were obtained commercially from ATCC. The TgMSH-1 deficient parasite strain was created by random insertional mutagenesis of RHΔ*hpt* parasites and is described in detail in Garrison et al. [9]. Normal culture medium was Dubelco's Modified Eagle Medium (DMEM) supplemented with 10% FBS, 2 mM L-glutamine and 100 units penicillin µg streptomycin per ml. For drug treatment experiments, normal culture medium was supplemented with monensin (Sigma), or monensin plus 3-methyladenine (Sigma). GFP-TgATG8 and GFP-TgATG8-G/A plasmid constructs [14] were a gift from S. Besteiro. To create the GFP-TgATG8 and GFP-TgATG8-G/A expressing parasite lines, RHΔ*hpt* parasites were electroporated with 30 µg of linearized plasmid. Parasites incorporating the plasmid were selected for by addition of 50 µg mycophenolic acid and 50 µg xanthine per ml of normal culture medium. After three rounds of selection individual parasite clones were established from each population by limiting dilution. GFP-positive clones were selected by fluorescence microscopy.

### Plaque assays

For the plaque assays 4×10^3^ parasites were added per well of 12-well tissue culture plates containing confluent HFFs. After 24 hours the media was removed and replaced by normal culture medium (controls), normal culture medium plus monensin (0.75 ng/ml), or normal culture medium plus monensin (0.75 ng/ml) and 3-methyladenine (10 µM final concentration). After 24 hours drug treatment, wells were washed and the medium replaced by normal culture medium. Plates were then incubated at 37°C for 6 days at which point the cultures were fixed in 100% methanol. Monolayers were then stained for 5 minutes with crystal violet to visualize and count the total number of plaques per well. The number of plaques in the testing conditions over that in the control conditions was presented as percentages. Results of all plaque assays are the average of 3 independent experiments ± standard deviation.

### Microscopy and immunofluourescence assays

Phase and immunofluorescence microscopy was performed using a Nikon Eclipse 80i microscope. Fluorescent images were deconvolved using NIS-Elements AR 3.0 software. All cells were fixed in 4% methanol-free formaldehyde (Thermo). Monoclonal antibodies 5F4 (anti-F1-ATPase beta subunit) and 11G8 (anti-ATrx1) {DeRocher, 2008 #1517}, to detect the mitochondria and the apicoplast, respectively, were gifts from P. Bradley. Localization of the plant-like vacuole was through the TgNHE3 polyclonal antibody [17]. Visualization of primary antibodies was by Alexa Fluor 594 goat anti-mouse (monoclonal primaries) or goat anti-guinea pig (polyclonal primary) IGG (Invitrogen). Cell monolayers were mounted on slides using Vectashield with DAPI (Vector). All counts of GFP-TgATG8 foci and mitochondria were done of at least 200 parasites. Results represent 3 independent experiments ± standard deviation.

### Electron Microscopy

HFFs infected with *T. gondii* were fixed in 2% parafomaldehyde/2% glutaraldehyde in 0.1 M cacodylate buffer followed by post fixation in 2% osmium tetroxide. Cells were dehydrated in an ethanol series followed by 100% acetone, infiltrated with Spurr's resin and polymerized overnight at 70°C. Thin sections (90 nm) were placed on formvar coated nickel grids and stained with 4% uranyl acetate and Reynold's Lead before viewing with JEOL 1200 EX located JEM transmission electron microscope at the Franceschi Microscopy and Imaging Center, Washington State University, Pullman Washington.

### Flow cytometry

Intracellular parasites were isolated by passage of host cells through a 30-gauge needle followed by filtration through a 3.0 µm pore-size membrane (Whatman). Parasites were then fixed in 70% ethanol and their DNA stained using 1 µm Sytox Green (Invitrogen) plus 50 units RNase A, 200 units RNase T1 (Ambion) per ml, in 50 mM Tris, pH 7.5. Samples were analyzed on a FACSAria flow cytometer (BD Biosciences). Results were analyzed using FlowJo software (Tree Star), and percentage of parasites in each phase of the cell cycle was estimated by gating. All assays were performed in triplicate and percentages of parasites in each phase of the cell cycle were compared for statistical significance between control and test groups by use of a two-tailed *t* test (P<0.05) using JMP software (SAS Institute).

## Results

### Monensin exposure causes reversible morphological changes in *T. gondii*

**Figure 1:**
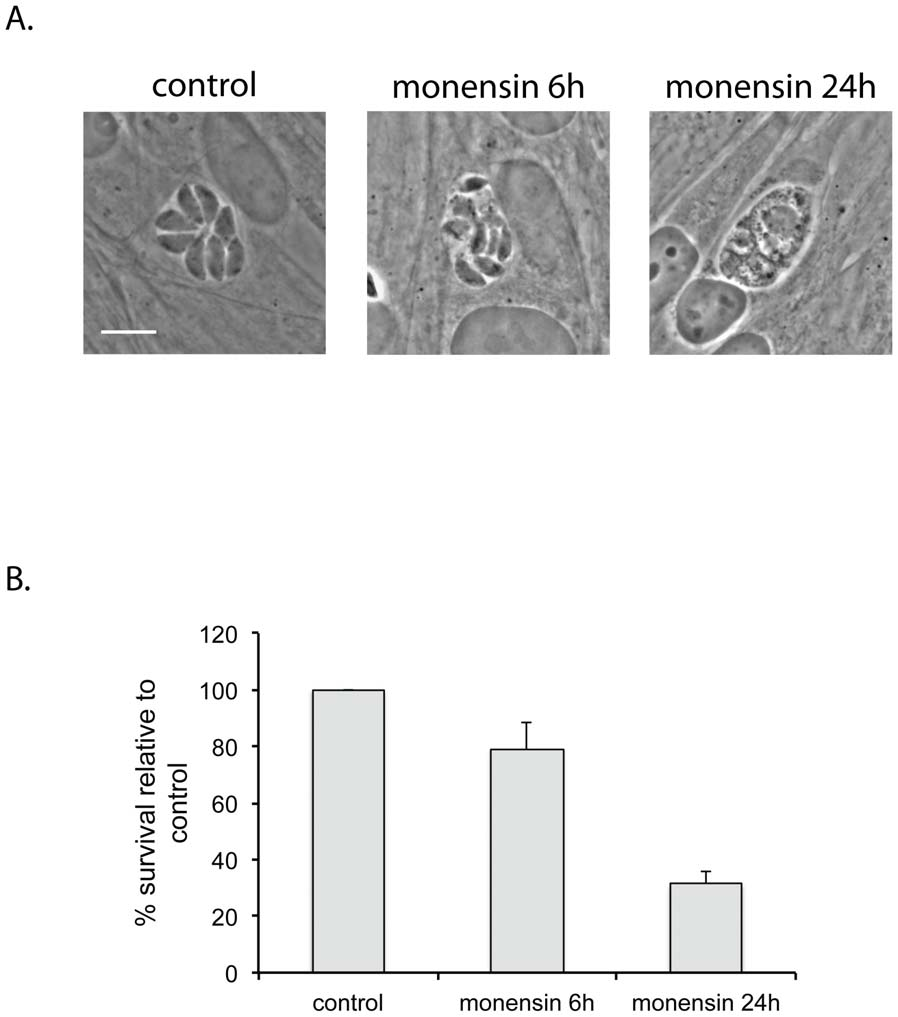
Effect of monensin on *T. gondii* is reversible. (A) Phase-contrast micrographs of intracellular *T. gondii* after exposure to 0.75 ng/ml monensin for 6 hours or 24 hours. By 24 hours 100% of parasites show the altered appearance pictured. Scale bar = 10 µm. (B) Survival of *T. gondii* after exposure to monensin. Parasites were exposed to 0.75 ng/ml monensin for 6 hours or 24 hours, after which the monensin was removed and parasites were allowed to form plaques. Data is expressed as % survival relative to control (no-monensin exposure) parasites. Control parasites are considered to have 100% survival. Each bar represents the mean value for three independent replicates. Error bar = 1 standard deviation.

In examining the effects of monensin on intracellular *Toxoplasma gondii* using phase-contrast microscopy, we noticed that monensin rapidly caused changes in the morphological appearance of the parasites (Fig. 1A). By 6 hours the parasites were becoming indistinct, suggesting that they were beginning to undergo lysis. By 24 hours of monensin exposure individual parasites were no longer visible in any vacuoles. Given the appearance of the parasites after 24 hours of monensin treatment, we assumed the vacuoles were filled with cellular debris and the parasites were dead. To confirm that the aberrant appearance of the individual vacuoles correlated with parasite death we used plaque assays to determine survival rates of *T. gondii* in human foreskin fibroblasts (HFF) exposed to monensin (0.75 ng/ml) for 6 hours, 24 hours, or continuously. Parasites continuously exposed to monensin never formed plaques (0.0±0% survival). However, when monensin was removed after 6 or 24 hours of exposure we observed 79.1±9.6% and 31.3±4.4% survival relative to controls (6 or 24 hours incubation with EtOH solvent alone), respectively (Fig. 1B). Thus the radical changes of parasite appearance observed in phase-contrast microscopy do not represent complete lysis of the parasites. Many parasites remain alive and can recover, even after 24 hours exposure to monensin, at which time point all parasites show the disrupted morphology shown in Figure 1A. Furthermore, this reversible effect of monensin treatment is dependent on the length of exposure to the drug.

**Figure 2:**
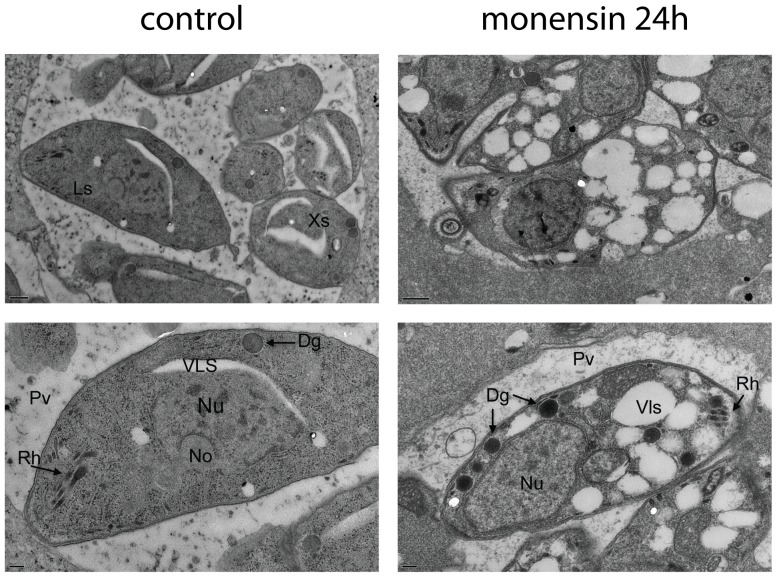
Monensin induces formation of vacuole like structures in *T. gondii*. Transmission electron micrographs of intracellular *T. gondii* after 24 hours exposure to 0.75 ng/ml monensin. Ls, longitudinal section through parasite; Xs, cross-section through parasite; Pv, parasitophorous vacuole; Rh, rhoptries; Nu, nucleus; No, nucleolus; Dg, dense granule; Vls, vacuole-like structure. Scale bar (bottom left) = 0.5 µM.

In order to determine what caused monensin-exposed parasites to change appearance in phase-contrast microscopy, we examined parasites under the same conditions using transmission electron microscopy. Parasites that were exposed to 0.75 ng/ml monensin for 24 hours were clearly intact within the parasitophorous vacuole and evidence of complete lysis was never observed (Fig. 2). However, these parasites appeared swollen, with little space between them, and had multiple intracellular vacuole-like structures. This often resulted in major distortions of the typical crescent shape of the parasite. This swelling and vacuolarization thus likely accounts for the loss of contrast and inability to distinguish individual parasites in the phase images. In treated parasites nuclei appeared intact. Similarly, the rhoptries (secretory structures found at the apical end of the parasite that function in invasion of host cells) [18] and dense granules (specialized secretory organelles) [19] appeared unchanged in treated parasites.

### Monensin induces reversible mitochondrial alteration in *T. gondii*

**Figure 3:**
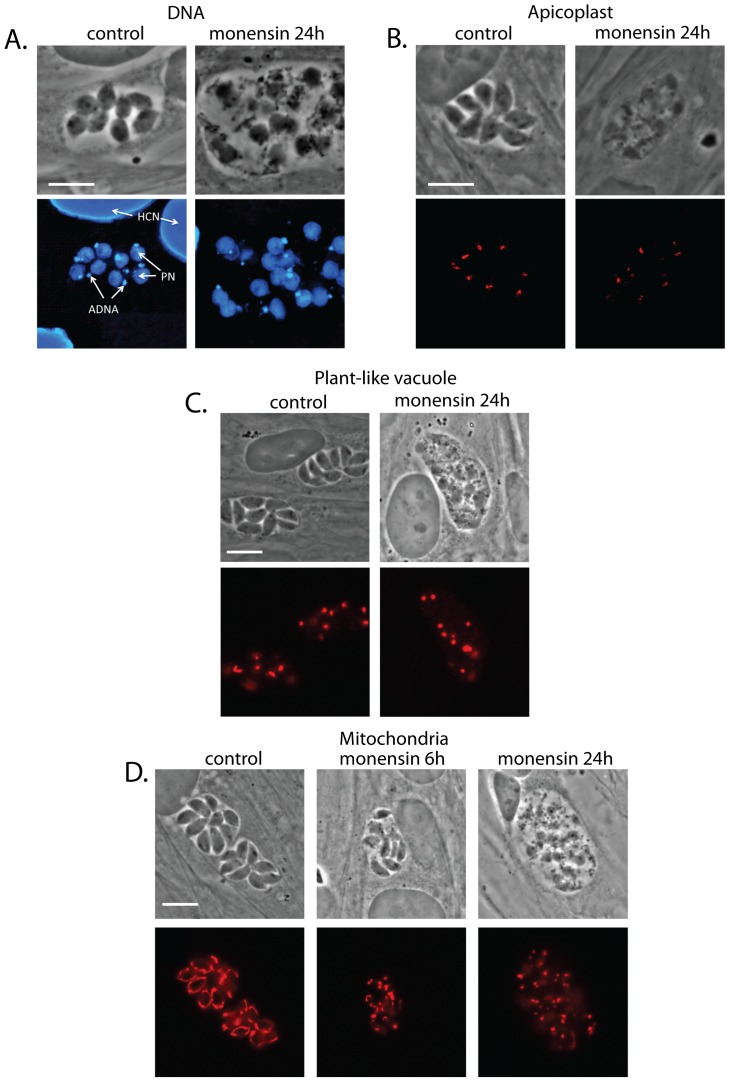
Monensin affects mitochondrial morphology. Phase-contrast and deconvolved immunofluorescence micrographs showing effect of monensin (0.75 ng/ml, 24 hours) on several *T. gondii* intracellular structures. (A) Phase contrast and DAPI staining of DNA showing parasite nuclei (PN) and apicoplast DNA (ADNA). HCN = host cell nuclei. (B) Phase contrast and immunofluorescence staining showing Atrx1 protein in the apicoplasts. (C) Phase contrast and immunofluorescence staining of TgNHE3 showing parasite plant-like vacuoles. (D) Phase contrast and immunofluorescence showing parasite mitochondria. Scale bars = 10 µm.

In order to further understand how monensin affects the morphology of the parasite, we stained intracellular parasites after 24 hours exposure to 0.75 ng/ml monensin with a series of antibodies that detect different *T. gondii* organelles (Fig. 3). As seen in the electron micrographs, the parasites' nuclei remained intact (Fig. 3A). The DNA associated with the apicoplast, a non-photosynthetic plastid present in many apicomplexan parasites, could also be clearly observed in both control and monensin-treated parasites. Confirming the DNA staining, an anti-apicoplast antibody showed that these organelles persisted and appeared normal (Fig. 3B). Staining of the parasites' plant-like vacuole with an antibody for the vacuole specific protein TgNHE3 [17] showed that the vacuoles did appear to persist after monensin exposure, which was not clear from the electron micrographs (Fig. 3C). In contrast to the other organelles, the mitochondria showed clear changes in morphology (Fig. 3D). In control parasites *T. gondii*'s single mitochondrion stained as a long, contiguous, ribbon-shaped structure. All monensin-exposed parasites (100±0%) had mitochondria that appeared as discontinuous punctae, suggesting mitochondrial dynamics are altered and fission occurs.

We were especially interested in monensin-mediated changes to the mitochondria, as we have previously shown that a *T. gondii* insertional mutant for a mitochondrial MutS homologue DNA repair enzyme is resistant to monensin [9]. Thus we suspected that the mitochondrion might be particularly important in susceptibility to monensin. Changes in mitochondrial morphology could be observed as early as 6 hours post-monensin exposure, with the mitochondrial ribbon of some parasites becoming more discontinuous and punctae beginning to form (Fig. 3D).

Because we had seen that many parasites can recover from monensin exposure, we also examined whether the effects of monensin on the parasites' mitochondrial morphology were also reversible. When parasites were exposed for 24 hours to monensin, then washed in normal culture medium and allowed 24 hours to recover, 54.3±16.0% of the parasites had normal mitochondrial morphology (as compared to 0±0% after 24 hours monensin exposure with no recovery). This correlated well with recovery of the overall shape of the parasite: 0±0% of the parasites had normal external morphology after 24 hours monensin exposure, while 54.0±15.1% had normal morphology after 24 hours recovery following 24 hours of monensin exposure. This further emphasizes that the extreme morphological changes induced by monensin do not necessarily result in the death of the parasites – they are survivable and reversible, at least for some of the parasites.

### Monensin induces autophagy in *T. gondii*

Our model for the action of monensin on *T. gondii* [10] hypothesized that parasites arrested in late S-phase would eventually die if the stimulus for cell cycle arrest was not reversed or repaired, akin to G_2_ checkpoints in other organisms. Typically, such G_2_ arrested cells would die by apoptosis [13]. We were not able to find typical hallmarks of apoptosis, such as DNA laddering, annexin V labeling, or caspase-like proteolytic activity, in monensin-exposed *T. gondii* (data not shown). Instead, several lines of evidence led us to suspect parasites exposed to monensin may initiate autophagy, which can represent an alternative cell-death pathway to apoptosis. We had previously found that monensin caused the upregulation of transcription of a ULK kinse (ATG1) homologue [10]. In addition, the morphology of mitochondria after monensin exposure looked very much like mitochondrial morphology in *T. gondii* reported by Ghosh et al. [15] after nutrient stress, which the authors believe is due to mitophagy, a specialized form of autophagy.

**Figure 4:**
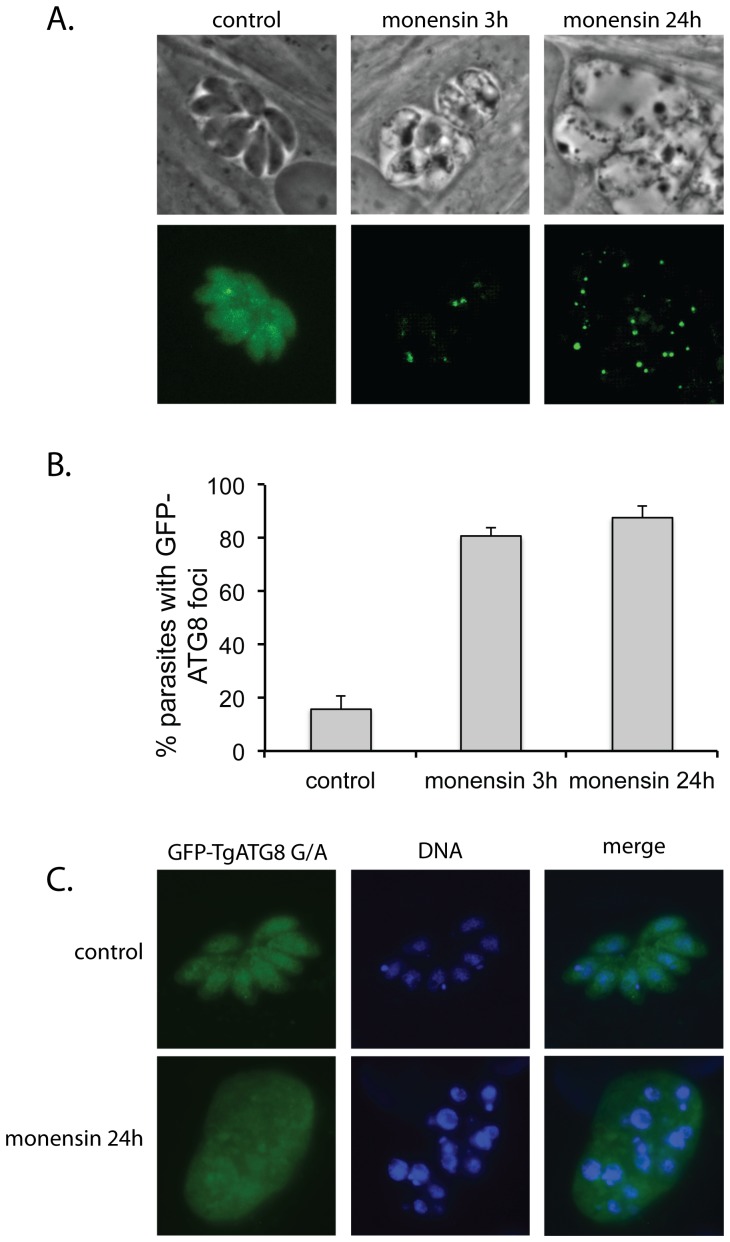
Monensin induces formation of GFP-ATG8 foci in intracellular parasites. (A) Phase contrast and deconvolved immmuofluorescent micrographs of *T. gondii* expressing GFP-tagged ATG8 after exposure to monensin (0.75 ng/ml) for 3 hours or 24 hours. (B) Percentage of parasites containing one or more GFP-ATG8 foci after exposure to monensin. Each bar represents the mean value for three independent replicates. Error bar = 1 standard deviation. (C) Deconvolved immmuofluorescent micrographs show that *T. gondii* expressing a GFP-tagged ATG8 in which the terminal glycine was replaced with an alanine (GFP-TgATG8-G/A) do not show formation of GFP-ATG8 foci even after 24 hours exposure to monensin (0.75 ng/ml).

A well-established method for detecting autophagy is to monitor the translocation of fluorescently labeled ATG8 from the cytoplasm to the forming autophagosome. Besteiro et al. [14] have established this technique for detecting autophagy in *T. gondii* using a strain of parasites expressing an exogenous copy of *T. gondii* ATG8 with a GFP label at its amino terminus (GFP-ATG8). The exogenous gene in this strain is under control of the strong tubulin promoter, which facilitates microscopic observation of the protein and detection of autophagy. Besteiro et al. [14] showed that parasites transfected with this plasmid normally produce a diffuse cytoplasmic signal. Upon induction of autophagy by nutrient stress or inducers such as rapamycin, the GFP-ATG8 becomes concentrated in one or more punctae, coinciding with the incorporation of ATG8 into the membrane of the autophagosome. We also saw that under control conditions *T. gondii* ATG8 with GFP fused at its amino terminus showed a diffuse, granular GFP signal throughout the cytoplasm (Fig. 4A). Also similar to Besteiro et al. [14], we found that a subset of these parasites contained foci of GFP (16.0±4.6%), but even these cells retained some diffuse cytoplasmic GFP signal. However, after exposure to monensin, the percentage of cells containing strong GFP foci increased, as did the number of foci per cell (Fig. 4A and 4B). This was a relatively rapid process, so that by 3 h post-monensin (0.75 ng/ml) exposure 80.7±3.1% of the parasites had GFP foci (Fig. 4A and 4B). In addition, the diffuse cytoplasmic GFP signal was typically greatly reduced or absent in these monensin-exposed parasites. This effect was still observed by 24 hours, with 87.7±4.5% of parasites showing GFP foci, and a general lack of diffuse cyctoplasmic signal (Fig. 4A and 4B). These results are similar to those of Besteiro et al [14], who found that for extracellular *T. gondii* the percentage of parasites with GFP-TgATG8 foci went from ∼15% in control medium to ∼79% after starvation by an 8 h incubation in saline. Thus monensin induces autophagy in intracellular *T. gondii*.

In order to confirm that GFP-positive punctae were in fact due to accumulation of labeled TgATG8 in developing autophagosomes, we also examined the effect of monensin on a parasite strain expressing an exogenous variant of the GFP-TgATG8 protein in which the C-terminal glycine was replaced with an alanine (GFP-TgATG8-G/A). Removal of the C-terminal glycine, which is necessary for lipidaption of ATG8 and consequent localization of the protein to the autophagasome, prevents formation of GFP-positive punctae even after exposure to autophagic inducers [14]. When *T. gondii* expressing GFP-TgATG8-G/A were exposed to 0.75 ng/ml monensin for 24 hours the GFP signal remained diffuse and cytoplasmic, and punctae were not formed (Fig. 4C), confirming that the effect of monensin on GFP localization was specific to the correct localization of the TgATG8, and not a non-specific (i.e. non-autophagy related) consequence of monensin exposure. It should be noted that the GFP-TgATG8-G/A protein was expressed as an exogenous copy, and although it was not properly localized to autophagosomes the endogenous copy of TgATG8 appeared to allow autophagy to proceed, as indicated by the presence of altered mitochondria in these parasites after monensin exposure.

**Figure 5:**
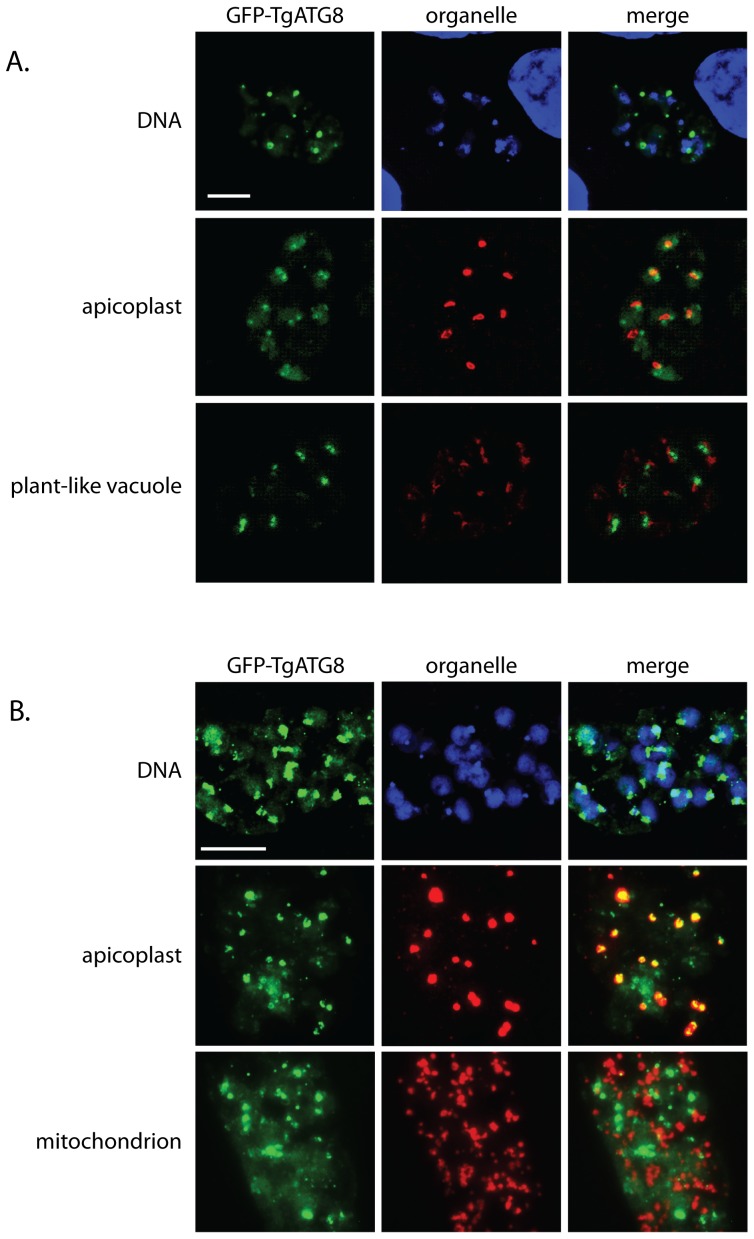
ATG8 colocalizes with the apicoplasts after prolonged monensin exposure. Deconvolved immunofluorescence micrographs showing relative localization of GFP-ATG8 and *T. gondii* intracellular structures after exposure to monensin (0.75 ng/ml). (A) 3 hours monensin exposure showing localization of GFP-ATG8 and DNA, apicoplasts, and plant-like vacuoles. (B) 24 hours monensin exposure showing localization of DNA, apicoplasts, and mitochondria. Scale bar = 10 µm.

We also used stained parasites in the absence and presence of monensin with the antibodies or stains we used in the previous section (mitochondria, apicoplasts, plant-like vacuole, DNA) to look for co-localization with GFP-TgATG8 (Fig. 5). At 3 hours monensin exposure, GFP-TgATG8 did not appear to co-localize with the plant-like vacuole (Fig. 5A) or mitochondria. However, the apicoplasts, and DAPI stained apicoplast DNA, did sometimes appear to co-localize with the GFP-positive foci (Fig. 5A). By 24 hours there was strong co-localization between the GFP signal and the apicoplast in nearly all cases (Fig. 5B), while the other antibody signals still did not colocalize with GFP. Thus GFP-TgATG8 appears to move to the apicoplast after localization of the protein in foci. However, it is not clear if the entire protein migrates to the apicoplast, or just a cleaved region containing the GFP tag. Nonetheless, it is clear that monensin causes a redistribution of ATG8 identical to what is seen with inducers of autophagy, strongly suggesting that this process is involved in the response to monensin treatment.

### The autophagy inhibitor 3-methyladenine inhibits formation of ATG8 punctae and mitochondrial morphological disruption in response to monensin

**Figure 6:**
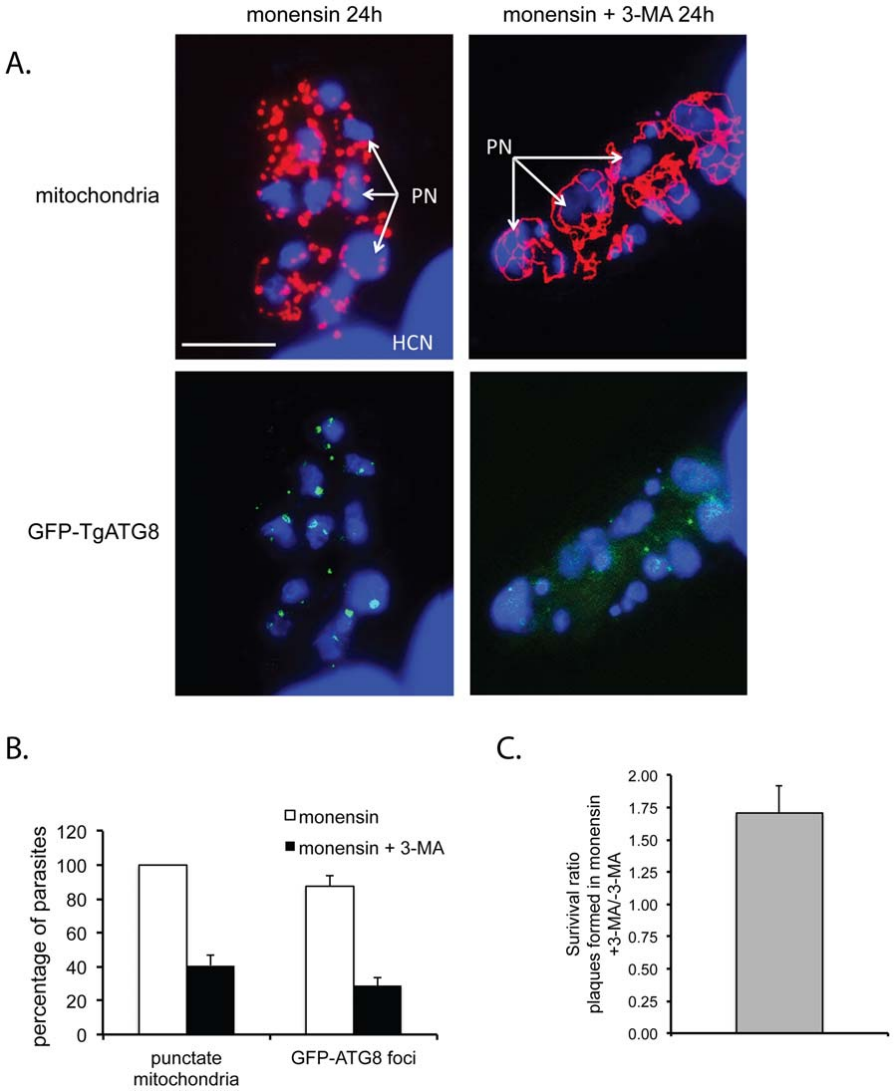
The autophagy inhibitor 3-methyladenine (3-MA) blocks autophagy and mitochondrial alteration induced by monensin. (A) Deconvolved fluorescence micrographs of intracellular *T. gondii* exposed to 0.75 ng/ml monensin or 0.75 ng/ml monensin plus 10 mM 3-MA for 24 hours. Red = mitochondria, green = GFP-ATG8, blue = DNA. PN, parasite nuclei; HCN, host cell nuclei. (B) Quantification of number of parasites positive for punctate mitochondria or GFP-ATG8 autophagosome foci after exposure to 0.75 ng/ml monensin for 24 hours (white bars) or 0.75 ng/ml monensin+10 mM 3-MA for 24 hours (black bars). Each bar represents the mean value for three independent replicates. Error bar equals the standard deviation. Scale bar = 10 µm. (C) The bar represents the ratio of number of plaques formed in 0.75 ng/ml monensin+10 mM 3-MA over that in only 0.75 ng/ml monensin. Error bar is the standard deviation.

3-methyladenine is widely used in experimental studies as a specific inhibitor of autophagy [20]. It has been specifically shown to inhibit autophagy in *T. gondii*, although this inhibition was only partial, indicating that 3-MA is not as effective an inhibitor of autophagy in *T. gondii* as it is in mammalian and yeast cells [14], [15]. We investigated whether adding 3-MA would affect monensin-induced GFP-TgATG8 relocalization and mitochondrial morphological disruption in *T. gondii*. Accordingly, GFP-TgATG8 expressing parasites were allowed to invade and develop in HFF monolayers for 24 hours, and then exposed to 0.75 ng/ml monensin or 0.75 ng/ml monensin plus 10 mM 3-MA for an additional 24 hours. Parasites were then immediately fixed and stained with an anti-mitochondrial antibody. Parasites exposed to monensin plus 3-MA showed a diffuse cyctoplasmic distribution of GFP-TgATG8, similar to parasites not exposed to monensin (Fig. 6A). The monensin plus 3-MA exposed samples also had fewer parasites containing GFP-positive foci than those exposed to monensin alone, with 28.8%±6.4% containing such foci, compared with 87.7±4.5% of those exposed to monensin alone (Fig. 6B). This effect was similar in nature but somewhat stronger than that observed by Besteiro et al [14], who found that ∼65% of extracellular parasites incubated for 8 h in HBSS plus 3-MA were positive for GFP-TgATG8 foci, compared to ∼85% of parasites incubated in HBSS alone. Thus 3-MA acts to inhibit monensin-induced autophagy. Furthermore, as seen in our previous assays, in parasites treated with monensin alone, 100±0% had punctate mitochondria. However, in parasites treated with monensin and 3-MA, only 40.8±6.0% of the parasites had punctate mitochondria, while 59.2±6.0% retained the normal ribbon-shaped mitochondrial morphology (Fig. 6B). This is similar to the effect observed by Ghosh et al [15], who found that after exposure to the autophagy-inducing drug rapamycin ∼80% of intracellular *T. gondii* showed punctate-staining mitochondria, but when co-incubated with rapamycin and 3-MA only ∼20% of parasites showed punctate mitochondria [15]. Thus, addition of an autophagy inhibitor significantly reduces the effect of monensin on mitochondrial morphology. This result also indicates that the observed alteration of the mitochondria is a direct consequence of monensin-induced autophagy.

### 3-methyladenine enhances parasite survival in the presence of monensin

To test whether autophagy was an integral part of monensin-induced death, we conducted plaque-based survival assays in the presence of 3-MA. Accordingly, parasites were allowed to invade and develop in HFF monolayers for 24 hours. The medium was then switched to complete cell culture medium containing, either 0.75 ng/ml monensin or 0.75 ng/ml monensin plus 10 mM 3-MA. After 24 hours, the cells were washed and returned to complete culture medium. In treatments with monensin and 3-MA there were 1.71±0.21 times the number of plaques formed in treatments with monensin alone, demonstrating that interfering with autophagy caused significant (determined by *t* test, *P*≤0.05) decrease in mortality observed in *T. gondii* as a result of monensin exposure (Fig. 6C).

### Monensin-induced autophagy is TgMSH-1 dependent

Previously we have shown that disruption of the locus for a *T. gondii* mitochondrial protein with homology to MutS homolog DNA repair enzymes (*TgMSH-1*) results in resistance to monensin [9]. In addition, monensin-mediated late S-phase cell cycle arrest is also TgMSH-1-dependent [10]. Therefore we also examined whether monensin-induced autophagy, measured by disruption of mitochondrial morphology, was downstream of TgMSH-1 function. TgMSH-1 deficient parasites were allowed to invade and develop in HFF monolayers for 24 hours, and then incubated in complete medium plus 0.75 ng/ml monensin for an additional 24 hours. Staining with an anti-mitochondrial antibody showed that after 24 hours exposure to monensin 81.7±11.2% of TgMSH-1 deficient parasites retained their normal mitochondrial morphology, compared to 0±0% of the parental strain. Thus monensin-induced autophagy appears to be TgMSH-1 dependent.

### 3-MA does not rescue monensin-induced late S-phase cell cycle arrest in *T. gondii*

**Figure 7:**
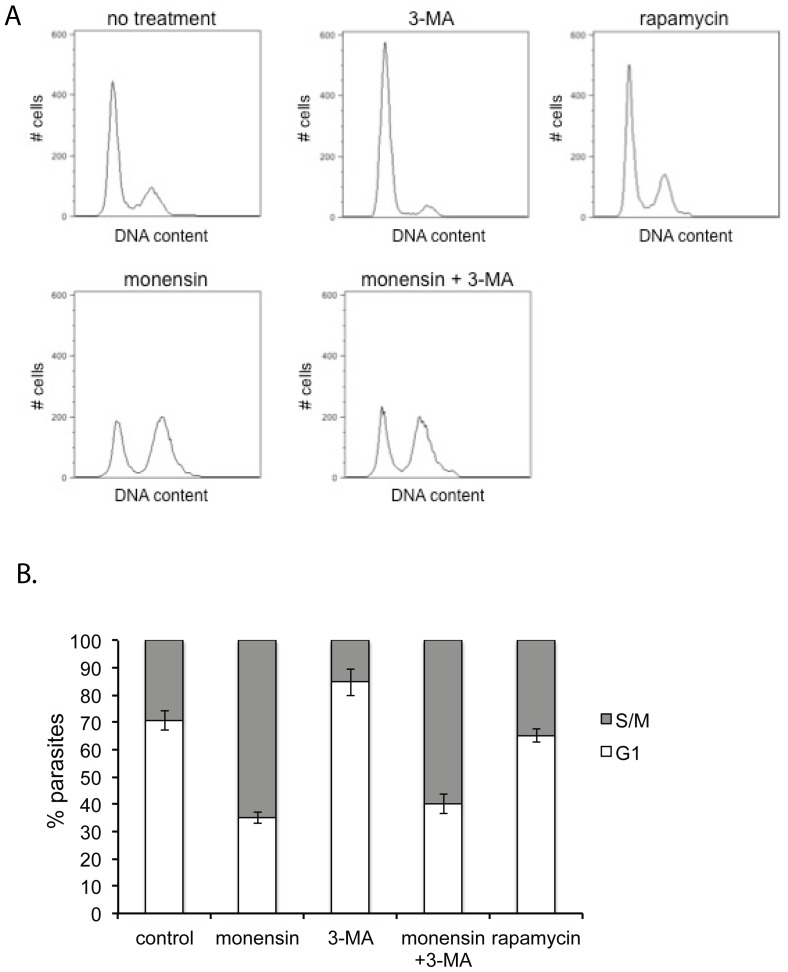
Rapamycin alone does not induce cell cycle arrest, nor does 3-MA rescue monensin-induced cell cycle arrest. Flow cytometry analysis of *T. gondii* cell cycle in response to rapamycin, monensin and 3-MA. Intracellular parasites were exposed to either normal culture medium or normal culture medium plus 0.75 ng/ml monensin, normal culture medium plus 10 mM 3-MA, normal culture medium plus 0.75 ng/ml monensin and 10 mM 3-MA, or normal culture medium plus 5 µM rapamycin. After 24 hours exposure, DNA content was measured by Sytox green staining. (A) Representative histograms are shown. Each histogram represents 10,000 total events. (B) Percentage ± standard deviation of parasites in G_1_ or S/M phases determined by gating for three separate experiments is indicated in the bar graphs.

Previously we have shown that monensin induces reversible arrest of the parasite cell cycle in late S-phase [10]. We examined whether monensin-induced autophagy appeared to be responsible for this S-phase arrest by examining the cell cycle of intracellular parasites that had been exposed to 0.75 ng/ml monensin, 10 mM 3-MA, or 0.75 ng/ml monensin +10 mM 3-MA, for 24 hours (Fig. 7). As previously shown [10], monensin caused an accumulation of parasites in late S-phase, with 65.0±2.0% of parasites in S-phase (compared to 29.3±3.5% in controls). 3-MA alone caused an accumulation of parasites in G_1_ of the cell cycle (84.7±5.0 in G_1_, compared to 70.7±3.5% in controls), similar to what was reported by Wang et al. [21]. However, exposure to monensin plus 3-MA resulted in a pattern of late S-phase arrest (40±3.6% in S-phase) that was not significantly different from exposure to monensin alone (determined by *t* test, *P*≤0.05) (Fig. 7). Thus 3-MA, although it acts to prevent monensin-caused death and alterations in mitochondrial morphology in *T. gondii*, does not rescue monensin-caused late S-phase cell cycle arrest. This suggests that monensin-induced autophagy is downstream of the induced cell cycle arrest, or that they are separate phenomena.

We further confirmed that cell cycle arrest does not appear to be a general consequence of authophagy by examining the effects of the autophagy-inducing drug rapamycin on the *T. gondii* cell cycle (Fig. 7). Rapamycin has been shown to induce autophagy in a wide variety of cell types, including in *T. gondii* [14], [15]. We found that intracellular parasites exposed to 5 µM rapamycin showed a cell cycle distribution of 65.3±2.5% in G_1_, not significantly different from that of parasites under normal conditions (determined by *t* test, *P*≤0.05).

## Discussion

Autophagy has been most thoroughly characterized as a cellular survival mechanism in response to starvation [22]. Indeed, previous reports of autophagy in *T. gondii* have been in response to nutrient stress induced by incubation of extracellular parasites in saline solution (Hank's buffered salt solution – HBSS) [14] or intracellular parasites in cell culture medium diluted with HBSS [15]. Here we show that autophagy is also induced by the anticoccidial drug monensin, and that this response represents a novel mechanism of parasite death in response to an antimicrobial drug.

In addition to its function in cell survival during stress conditions, it has been proposed that autophagy can act as a distinct method of cell death, termed autophagic cell death, although the concept is still frequently debated [16]. In support of autophagy being a causative agent of cell death, it has been demonstrated that in some instances blocking autophagy can maintain cell viability [23]. Given that *T. gondii* has not been shown to undergo apoptosis, Ghosh et al. [15] hypothesized that autophagy may replace apoptosis as a cell death pathway in the parasite. However, experimental evidence was limited to showing that inhibiting autophagy allowed starvation-treated parasites to invade cells, and nutrient stress is not typically considered an initiator of apoptosis. Here we show that inhibition of autophagy allows parasites to survive lethal dose of monensin, a direct demonstration that autophagy can act as a cell death mechanism in *T. gondii*. In addition, our results suggest that autophagy occurs after prolonged arrest at a cell cycle checkpoint, a condition that would typically cause cells to undergo apoptosis [13]. Importantly, parasites treated with the autophagy inhibitor 3-MA showed not only decreased autophagy, but also enhanced survival in the presence of monensin. This effect was not complete, but 3-MA is not as effective in inhibiting autophagy in *T. gondii* as it is in mammalian and yeast cells [14], and in fact *T. gondii* shows a susceptibility to 3-MA-mediated autophagy suppression much more akin to that of plants [24]. Although 3-MA does not provide complete inhibition of autophagy in *T. gondii*, the present study does provide experimental evidence that autophagy may be an important cell death mechanism in lieu of apoptosis.

Like Besteiro et al. [14], we found that even under normal conditions, some portion (∼16% in our study) of the parasites appeared to be undergoing autophagy, as indicated by GFP-TgATG8 localization in punctae. Besteiro et al. [14] correlated this GFP-positive punctae formation with specific stage of the cell cycle, and found that it seemed to occur especially in cells that are actually in the process of cytokinesis, suggesting that autophagy may be involved in recycling some components of mother cells during the parasite's division process of endodyogeny. In any case, we found that after monensin exposure there was a sharp increase in the number of parasites showing GFP-positive punctae, and a concomitant decrease in overall cytoplasmic GFP signal. Although monensin disrupts the parasite's cell cycle, it causes an increase in the proportion of parasites in late S-phase of the cell cycle, and actually decreases the number of parasites that progress to cytokinesis [10]. Thus any potential association of autophagy with cytokinesis is unrelated to the increase in autophagy we observed in parasites that are arrested in late S-phase by monensin.

Despite monensin's effect on the parasites' mitochondria, we could not co-localize GFP-TgATG8 punctae with the punctate signal seen in mitochondria (as a result of immunofluorescence staining) after monensin exposure. Formation of GFP-TgATG8 punctae occurred much more quickly – by 3 hours monensin exposure ∼80% of parasites were positive for these punctae. However, formation of a punctate signal in mitochondria was just beginning by 6 hours. Even after prolonged exposure to monensin we did not observe co-localization of GFP-TgATG8 and mitochondrial signals. Instead we found that after such exposure (24 hours), GFP-ATG8 punctae persisted and there was close co-localization between GFP-TgATG8 and the parasites' apicoplasts. Besteiro et al. [14] also reported the presence of relatively large GFP-TgATG8 positive vesicles in the region of the apicoplast in a subset of extracellular parasites. In yeast, ATG8 is involved in formation of the autophagosome precursor, the autophagophore, but is subsequently released and recycled to the cytoplasm during maturation of the autophagosome (although some ATG8 can apparently become trapped in the autophagosome) [25]. It is not clear if this process is what is occurring in *T. gondii* after monensin exposure, and how this relates to our observation of association between GFP-TgATG8 punctae and apicoplasts after prolonged exposure to monensin. Monensin has also been shown to inhibit lysosomal degradation of proteins in mammalian cells by altering lysosomal pH [26]. It is not known whether lysosomal degradation of proteins in *T. gondii* is affected by the concentrations of monensin used in this study. Monensin concentrations necessary to affect lysosomal protein degradation in mammalian cells are several thousand-fold greater than the monensin concentration used in this study, although *T. gondii* is much more sensitive to monensin than mammalian cells. Thus, persistence of GFP-TgATG8 punctae may be due to inhibition of normal protein degradation. However, methylnitrourea (MNU), which like monensin induces a TgMSH-1-dependent late S-phase arrest in *T. gondii* [10], also induces the same pattern of GFP-TgATG8 punctation (Lavine and Arrizabalaga, unpublished observations), even though it is a dissimilar compound with no known effect on lysosomal degradation. Even if lysosomal function is impaired, it is not clear why GFP-TgATG8 should co-localize with the apicoplast. The *T. gondii* apicoplast is a relict plastid that seems to have multiple metabolic functions, including synthesis of isoprenoids, fatty acids, and heme [27]. Our results bring up the possibility that the apicoplast may also function in the recycling/regulation of TgATG8, although further research will be necessary to establish a more definitive connection.

Our results show that the organelle most rapidly affected by exposure to monensin is the mitochondrion. *T. gondii* exposed to monensin show pronounced alterations in mitochondrial morphology as visualized through immunofluorescence staining, with the normally ribbon-shaped mitochondria assuming first an appearance of “beads on a string”, and then becoming a series of distinct punctae. This superficially suggests that the mitochondria are fragmenting, although they may well remain intact after monensin exposure, despite showing very altered staining patterns in immunofluorescence assays. This alteration of mitochondrial morphology appears to be a consequence of autophagy, as it can be rescued by inhibition of autophagy with 3-methyladenine. The changes in mitochondrial morphology induced by monensin look very similar to those reported by Ghosh et al. [15] in response to nutrient stress caused by maintaining parasites and host cells in dilute culture medium. However Ghosh et al. [15] found that parasites with punctate mitochondria could not recover normal morphology and were not viable. In the case of monensin exposure, we found a significant proportion of the parasites could recover mitochondrial morphology and remain viable, if the monensin was removed after 24 hours. Thus it is unclear if this indicates that mitochondrial morphology changes due to monensin or dilute culture medium represent qualitatively different responses, or whether the difference is simply due to the dose or severity of stressor. Further research will be needed to tell precisely the effect of monensin on the mitochondrion. In any case, given what is known about autophagic cell death, we would not expect induction of autophagy to lead irreversibly to death, but instead would expect the effects to be reversible for some temporal and dose-dependent conditions [16], [23].

We have previously shown that monensin induces a cell cycle arrest of parasites during late S-phase but not bradyzoite gene expression [10]. The specific signaling mechanism for autophagy induction by monensin is unclear, but several of our results indicate that such induction may occur downstream of monensin induced cell cycle arrest. Rapamycin, a known initiator of autophagy in *T. gondii* [14], [15], did not alter the parasite's cell cycle, indicating that late S-phase arrest is not a general consequence of autophagy. In addition, blocking autophagy with 3-MA did not rescue parasites from late S-phase arrest, although it rescued mitochondrial phenotype and enhanced survival. Thus monensin-induced autophagy also does not appear to be upstream of monensin-induced cell cycle arrest. Methylnitrourea, which induces late S-phase arrest [10], also induces autophagy, as determined by accumulation of GFP-TgATG8 punctae and changes in mitochondrial morphology (Lavine and Arrizabalaga, unpublished observations). Furthermore, parasites deficient in the mitochondrial DNA repair enzyme TgMSH-1 are resistant to both cell cycle arrest [10] and monensin-induced autophagy (as measured by alterations in mitochondrial morphology). This means that autophagy either occurs downstream of TgMSH-1-mediated cell cycle arrest, or that monensin-induced autophagy and cell cycle arrest are parallel phenomena, both mediated by TgMSH-1 but otherwise independent. Currently, based on analogy to other cell types, we favor the hypothesis that monensin-induced autophagy occurs as a downstream consequence of monensin-induced cell cycle arrest. Certainly, in other cell types MutS homologues have been shown to mediate both DNA repair and detection of DNA damage, which initiates a signal transduction cascade leading to cell cycle arrest, typically in G_2_ of the cell cycle [11], [12]. We infer the function of TgMSH-1 based on homology to sequences of other *MSH* genes from other organisms [9]. The TgMSH-1 dependence of late S-phase arrest in *T. gondii* exposed to monensin is consistent with such a conserved function. Given the emerging knowledge from other cell types of the role mitochondria play in regulating autophagy [28], it is certainly possible that TgMSH-1, which is localized to the parasite's mitochondrion, directly regulates both the cell cycle and autophagy. Perhaps monensin could have effects on host cell nutrient availability that cause nutrient stress in *T. gondii*, inducing TgMSH-1-mediated autophagy. Although monensin can have effects on mammalian cells, including vesicle transport [29], lysosomal pH [26], and mitochondrial damage [30], these effects occur at drug concentrations hundreds to thousands times greater than the monensin concentration used in this study. Further research will be necessary to determine precisely how monensin activates autophagy in *T. gondii*. Currently, we believe that indirect regulation of autophagy through direct regulation of the cell cycle is the more parsimonious and likely scenario.

Thus, we hypothesize that TgMSH-1-mediated cell cycle arrest serves as a signal to initiate autophagy, akin to MSH-mediated G_2_ arrest serving as a signal to initiate apoptosis in other cell types. In either case, our results point to the potential importance of autophagy as a mechanism of drug-induced cell death in *Toxoplasma gondii*, and potentially in other apicomplexan parasites as well. This opens up intriguing possibilities that a better understanding of autophagy in pathogenic protists can have important implications in mechanisms of drug resistance and in the design and understanding of new anti-parasitic drugs.
